# Numerical modeling of rapidly varying flows using HEC-RAS and WSPG models

**DOI:** 10.1186/s40064-016-2199-0

**Published:** 2016-05-20

**Authors:** Prasada Rao, Theodore V. Hromadka

**Affiliations:** Civil and Environmental Engineering Department, California State University, Fullerton, CA 92831 USA; Department of Mathematical Sciences, United States Military Academy, West Point, NY 10996 USA

**Keywords:** Open channel, Roughness, Hydraulic jump, Froude number

## Abstract

The performance of two popular hydraulic models (HEC-RAS and WSPG) for modeling hydraulic jump in an open channel is investigated. The numerical solutions are compared with a new experimental data set obtained for varying channel bottom slopes and flow rates. Both the models satisfactorily predict the flow depths and location of the jump. The end results indicate that the numerical models output is sensitive to the value of chosen roughness coefficient. For this application, WSPG model is easier to implement with few input variables.

## Background

Modeling one dimensional rapidly varying flow in open channels has drawn the attention of many researchers. Referred to as hydraulic jump, it serves as a transition region between supercritical and sub-critical flows. Numerical formulations used for solving the one dimensional unsteady flow equations are considered satisfactory if they can capture the jump location and the flow depths. While initial efforts for solving the flow equations used standard explicit and implicit finite difference (Fennema and Chaudhry 1986) and finite element (Katapodes and Strelkoff 1988) formulations, the reliability in using high resolution numerical schemes was first detailed in the work of Shu and Osher (1988). The primary advantage in using high resolution schemes lies in their ability to generate a numerical solution that is devoid of any oscillations, which is a characteristic feature of second and higher order accurate finite difference schemes. Most of the published works (Birman and Falcovitz 2007; Ying et al. 2004; Venutelli 2004) over the last few years have highlighted the application of a variant of high resolution schemes. In this work, we take two popular hydraulic models and test their ability for modeling hydraulic jump over a variable bottom slope channel.

Given the apparent validity of Moore’s Law regarding the doubling of computer capabilities every 18 months (for example, refer to various references including the web video by Phil Roe entitled “Colorful Fluid Dynamics: Behind The Scenes”, among others), the application of computational engineering mathematics to fluid and flow energy transport problems has become commonplace. Additionally, computer software investments are increasingly focused towards pre- and post-processors that simplify computer program input efforts and enhance computational outcome visualization to the extent that the end-user’s analysis energy is increasingly focused towards enabling computer program input–output capability rather than validating and verifying computational accuracy in solving the governing flow equations. As a result, complex computer program capabilities may be applied towards computationally modeling boundary value problems that may be poorly posed in the numerical approximation sense. However, the computer program computational procedure may still produce modeling results that are embraced by the end-user as being accurately modeled. An approach to assessing the modeling accuracy is to apply multiple computer models to the target problem and compare the computational results. Differences in modeling outcomes between computational models may be a signal of various complications with the computational model itself or with the problem definition, among other issues. In this paper, we identify a computationally challenging flow problem that commonly occurs in flood control design and planning; namely, the problem of predicting the location and natures of a hydraulic jump. Other such computationally challenging benchmark level situations are of interest and will be the subject of future evaluations, including flow over a “hump” in a prismatic channel, and the flow regime involved with a junction of two flow paths. These benchmark situations, among others, may provide insight to computational model users as to the veracity of such computational modeling predictions. The validation of computational models stems ultimately from laboratory or measured flow situation data and, therefore, comparison of such modeling outcomes to measured data are of high value. In this paper, computational outcomes are compared with laboratory measured data for hydraulic jump scenarios.

Hydrologic Engineering Center-River Analysis System (HEC-RAS) is a one dimensional model that was developed by the U.S. Army Corps of Engineers Hydrologic Engineering Center (2005). It has found wide application for analyzing flow in rivers and in flood plain studies. The Water Surface Pressure Gradient (WSPG) model was initially developed by the Los Angeles County Flood Control District. Subsequent enhancements has made it popular among the southern California flood control districts (Civil Design Corporation 2010).

Literature review points out to the popularity of HEC-RAS model for analyzing flow in rivers with hydraulic jump. While a couple of related papers are referenced here, more references can be found in these cited works. Lee et al. (2006) used HEC-RAS model to simulate flooding in a river basin across a Typhoon. The effect of bridge blockage and over bank flow on water stage variation was modeled. Horritt and Bates (2002) comparatively assessed the predictive power of flood inundation models produced with HEC-RAS, LISFLOOD-FP, and TELEMAC-2D to determine the models suitability for hazard assessment. By analyzing two flood events, on the same 60 km reach of the river Severn, in the UK, they found that both HEC-RAS and TELEMAC-2D, after suitable calibrations, give good predictions of the inundated area. Jowhar and Jihan (2012) used HEC-RAS to predict the water surface profile, determine the location of the hydraulic jump, and establish the head discharge relationship of the trapezoidal profile weir. Endreny et al. (2011) used HEC-RAS to predict the steady state hydraulic jumps across river steps.

In this work, the model results are compared to the experimental data that was generated at the Hydraulics laboratory in California State University, Fullerton. This data was obtained for a variety of steady state flow conditions. Readers who would like to have the complete data are encouraged to correspond with the first author.

## Experimental setup

**Fig. 1:**
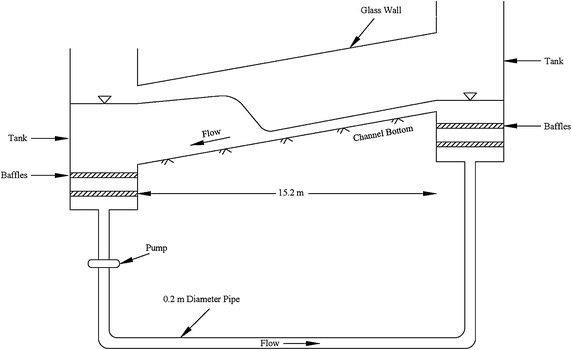
Definition sketch of the experimental facility

A review of the published experimental data did not yield any satisfactory complete data set that can be used for this investigation. The closest published data set was that of Gharangik and Chaudhry (1991) who conducted hydraulic jump experiments across a horizontal channel. Since both WSPG and HEC-RAS models require a specified channel bottom slope, we could not use their data. Our correspondence with few of our colleagues did not generate any new leads. Hence, experiments were conduced in the Hydraulics Laboratory at California State University, Fullerton. The schematic of the experimental facility is shown in Fig. 1.

The 15.2 m (50 ft) open channel has glass walls along the sides. The channel cross section is rectangular with a width of 0.46 m. The channel bottom slope can be adjusted by raising the channel at the upstream end. The water circulates in the test facility and the discharge can be varied by changing the pump speed. The tests were conducted for three bottom slopes, across a range of discharge values. The channel bottom roughness varied from 0.007 to 0.011. Table 1 lists the experimental data for all the test runs. For each run, flow measurements were taken after steady state flow conditions have been established.

**Table 1:** Experimental flow data

Data set	Bed slope	Y (m) at X = 0	Y (m) at X = 15.2 m	Q (m^3^/s)	Y_1_ (m)	Y_2_ (m)	F_1_	F_2_	X_1_ (m)	X_2_ (m)
1	0.01	0.07	0.23	0.047	0.07	0.13	1.59	0.7	7.62	7.93
2	0.01	0.10	0.21	0.054	0.08	0.14	1.74	0.68	9.75	10.2
3	0.012	0.04	0.24	0.036	0.04	0.11	3.37	0.66	6.71	6.86
4	0.012	0.07	0.23	0.045	0.06	0.15	2.14	0.54	8.84	9.14
5	0.012	0.08	0.23	0.051	0.07	0.13	2.03	0.76	10.06	10.21
6	0.02	0.05	0.28	0.034	0.04	0.12	2.76	0.6	7.8	8.05
7	0.02	0.06	0.27	0.040	0.05	0.14	2.05	0.53	9.32	9.51
8	0.02	0.07	0.27	0.049	0.06	0.17	2.22	0.49	10.52	10.67

Y_1_ and Y_2_ are the flow depths before and after the hydraulic jump. F_1_ and F_2_ are the respective Froude numbers. X_1_ and X_2_ are the distances from upstream corresponding to Y_1_ and Y_2_

## Model theory

The HEC-RAS model solves the one dimensional unsteady flow equations. These equations, in conservation form, can be written as (USACE 2005)

1 $$\frac{\partial A}{\partial t} + \frac{{\partial \left( {\Phi Q} \right)}}{{\partial X_{c} }} + \frac{{\partial \left[ {\left( {1 - \Phi } \right)Q} \right]}}{{\partial X_{f} }} = 0$$

2 $$\frac{\partial Q}{\partial t} + \frac{{\partial \left( {\Phi^{2} Q^{2} } \right)}}{{\partial X_{c} }} + \frac{{\partial \left[ {\left( {1 - \Phi } \right)^{2} Q^{2} /A_{f} } \right]}}{{\partial X_{f} }} + gA_{c} \left[ {\frac{\partial Z}{{\partial X_{c} }} + S_{fc} } \right] + gA_{f} \left[ {\frac{\partial Z}{{\partial X_{f} }} + S_{ff} } \right] = 0$$

where $$\Phi = \frac{{K_{c} }}{{K_{c} + K_{f} }}$$; $$K = \frac{{A^{5/3} }}{{nP^{2/3} }}$$; $$S_{fc} = \frac{{\Phi^{2} Q^{2} n_{c}^{2} }}{{R_{c}^{4/3} A_{c}^{2} }}$$; $$S_{ff} = \frac{{(1 - \Phi )^{2} Q^{2} n_{f}^{2} }}{{R_{f}^{4/3} A_{f}^{2} }}$$.

Subscripts *c* and *f* indicate the channel and floodplain, Q is the flow rate, X_c_ and X_f_ are the distances along the channel and floodplain, A_c_ and A_f_ are the flow areas in channel and flood plain, R_c_ and R_f_ are the hydraulic radius for the channel and floodplain, P is the wetted perimeter, $\Phi$ is the flow partitioning factor between the channel and floodplain and n is the Manning’s roughness coefficient. The model solves the above equations using a four-point implicit scheme. The model version 3.1.3 was used in this work.

The WSPG model solves the Bernoulli energy equation between any two sections, using the standard step method (Civil Design Corporation 2010). The program computes uniform and non-uniform steady flow water surface profiles. As part of the solution, it can automatically identify any hydraulic jump in the channel reach. The model version 12.99 was used in this work.

## Results

Table 1 lists the required flow data for the simulations. Column 2 is the bed slope of the channel. The maximum bed slope that was possible for this experimental setup was 0.02. Columns 3 and 4 are the flow depths at the upstream and downstream end of the channel. The measurements were taken after the flow reached a steady state condition. Column 5 is the flow rate. Columns 6 and 7 are the flow depths in the vicinity of the hydraulic jump. Even though, the flow profile had some fluctuations, they were minimal. However, more measurements were taken in this vicinity, to capture these small variations. Columns 8 and 9 are the Froude numbers $\left( F_{r} = \frac{V}{{\sqrt {gy} }} \right)$, before and after the jump. Columns 10 and 11 are the locations of the start and end of hydraulic jump, from the upstream end.

**Fig. 2:**
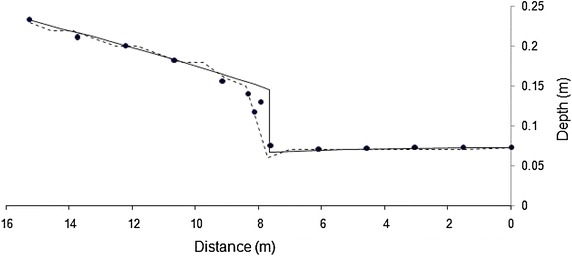
Comparison of the model results for Data Set 1. *Filled circle* experimental, *solid line* WSPG (n = 0.009), *dotted line* HEC RAS (n = 0.007)

**Fig. 3:**
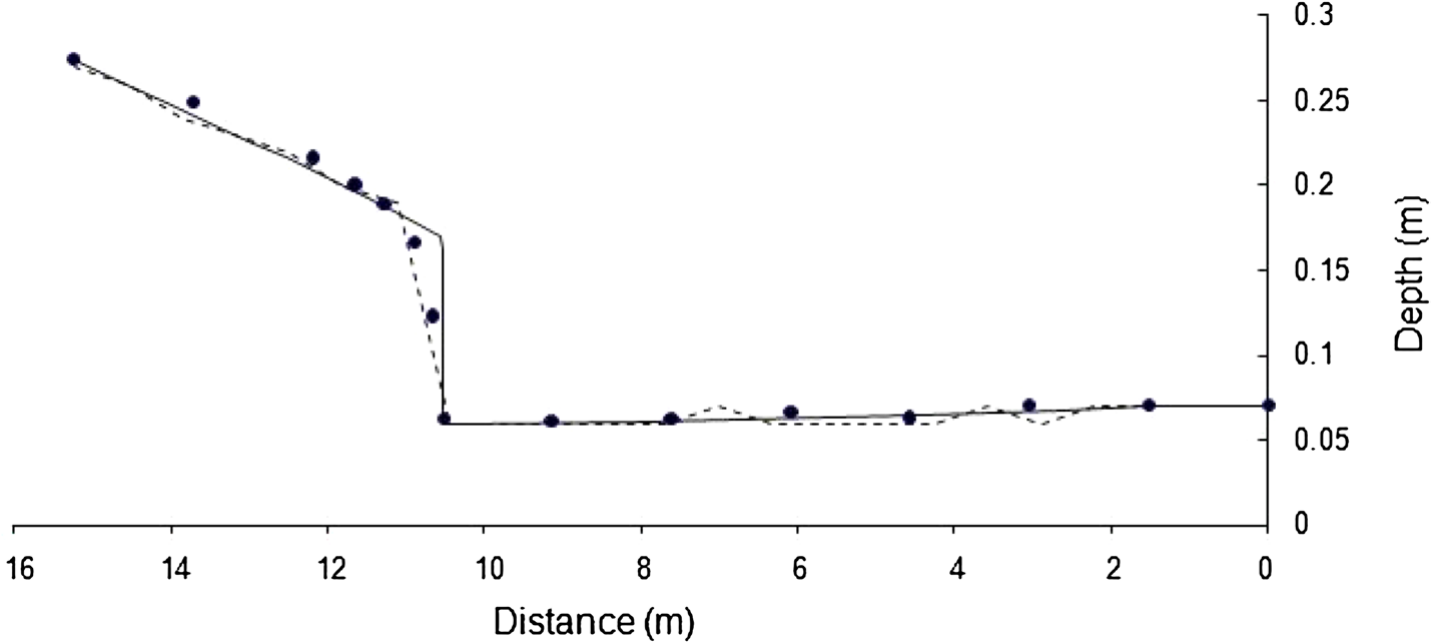
Comparison of the model results for Data Set 8. *Filled circle* experimental, *solid line* WSPG (n = 0.01), *dotted line* HEC-RAS (n = 0.008)

**Fig. 4:**
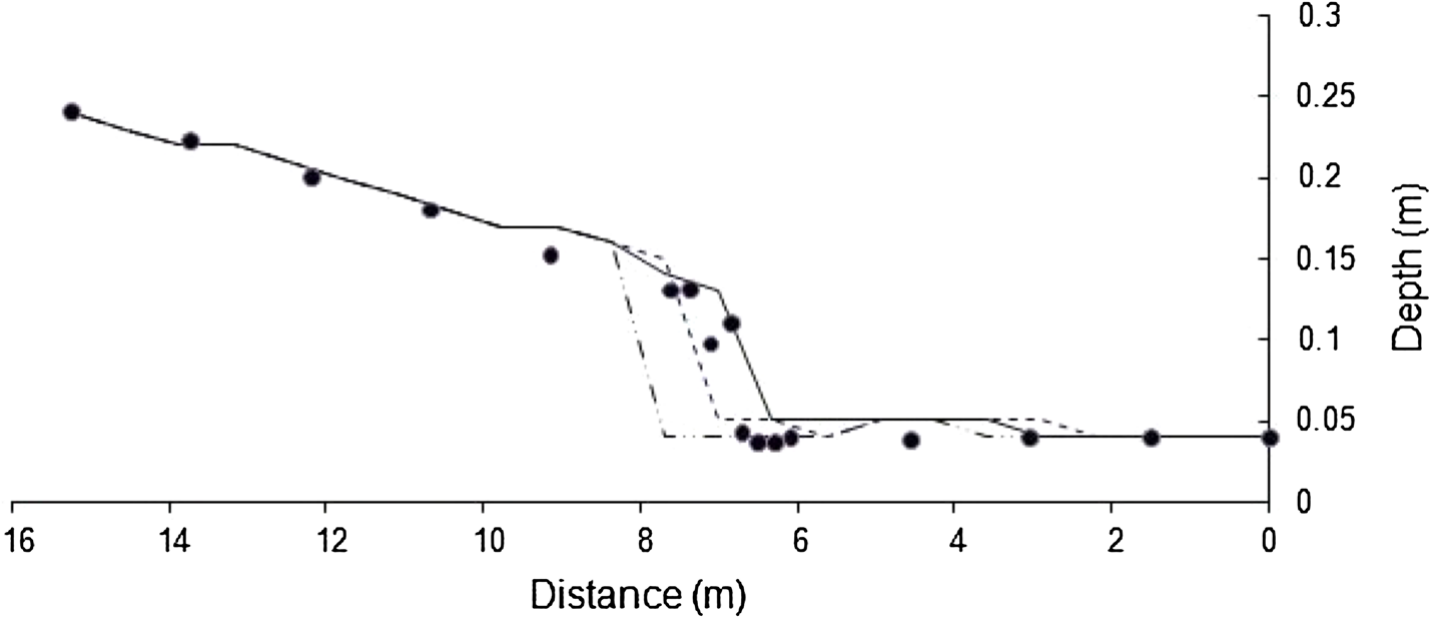
Effect of roughness value on HEC-RAS solution for Data Set 3. *Filled circle* experimental, *solid line* n = 0.01, *dotted line* n = 0.012, *dashed line* n = 0.009

**Fig. 5:**
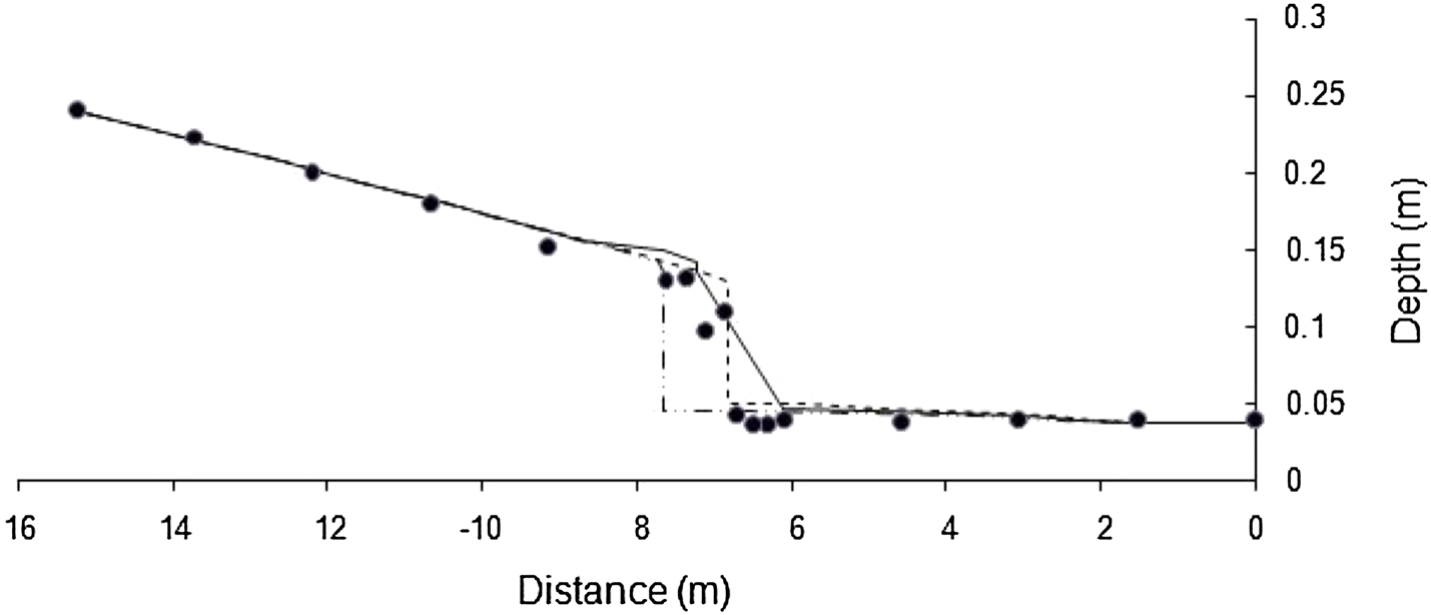
Effect of roughness value on WSPG solution for Data Set 3. *Filled circle* experimental, *solid line* n = 0.01, *dotted line* n = 0.011, *dashed line* n = 0.009

To conserve space, one result for each bed slope is presented in Figs. 2 and 3. Both models simulate the flow profiles satisfactorily. WSPG solution is devoid of any numerical oscillations and the shock front has the characteristics of that obtained using a high resolution numerical scheme. HEC-RAS solution has numerical oscillations, which is a characteristic feature of any standard implicit scheme. No effort was made in tuning the parameters in HEC-RAS model as these oscillations did not pose any stability problem. While WSPG simulates the hydraulic jump as part of the solution, in HEC-RAS, mixed-flow option needs to be selected. The location of the jump was observed to be sensitive to the value of roughness coefficient. For results in Figs. 2 and 3, an optimal roughness value for each model (which generated the solution close to the experimental data) was used. The roughness values are indicated in the figures. The trends of these results were valid for all the other data sets. Figures 4 and 5 illustrate the effect of roughness value on the jump location for both the models. The results indicate that while roughness value does not affect the flow depths, it has an impact on the jump location.

## Conclusions

HEC-RAS and WSPG models were used to simulate hydraulic jump in rectangular open channel. The model results were compared with the experimental data set, that was obtained across channel slopes. Salient observations are (1) Both models predict the jump characteristics satisfactorily (2) The roughness value impacts the location of the jump. Its affect on the flow depth is minimal and (3) For this application, WSPG model is easier to implement and requires less input information.
